# Oxytetracycline Pharmacokinetics After Intramuscular Administration in Cows with Clinical Metritis Associated with *Trueperella Pyogenes* Infection

**DOI:** 10.3390/antibiotics9070392

**Published:** 2020-07-09

**Authors:** Rositsa Mileva, Manol Karadaev, Ivan Fasulkov, Tsvetelina Petkova, Nikolina Rusenova, Nasko Vasilev, Aneliya Milanova

**Affiliations:** 1 Department of Pharmacology, Animal Physiology and Physiological Chemistry, Faculty of Veterinary Medicine, Trakia University, 6000 Stara Zagora, Bulgaria; rositsamileva88@gmail.com (R.M.); ts_petkova87@abv.bg (T.P.); 2 Department of Obstetrics, Reproduction and Reproductive Disorders, Faculty of Veterinary Medicine, Trakia University, 6000 Stara Zagora, Bulgaria; karadaev@abv.bg (M.K.); i.fasulkov@gmail.com (I.F.); nasvas@abv.bg (N.V.); 3 Department of Veterinary Microbiology, Infectious and Parasitic Diseases, Faculty of Veterinary Medicine, Trakia University, 6000 Stara Zagora, Bulgaria; n_v_n_v@abv.bg

**Keywords:** oxytetracycline, pharmacokinetics, cows, clinical metritis, *Trueperella pyogenes*

## Abstract

Systemic therapy with oxytetracycline is often used for treatment of clinical metritis although data about its penetration into the uterus and uterine secretion are lacking. Uterine secretions and milk from six cows with clinical metritis were collected for microbiological assay. The animals were treated intramuscularly with long-acting oxytetracycline (20 mg/kg) and samples of plasma, milk and uterine secretions were collected for determination of the antibiotic concentrations by HPLC-PDA analysis. Pharmacokinetics of the antibiotic and in silico prediction of its penetration into the uterus were described. *Trueperella pyogenes* with MIC values of 16–64 µg mL^−1^ was isolated (*n* of cows = 4) from uterine secretions. Oxytetracycline showed fast absorption and penetration in the uterine secretions and milk. No change of withdrawal time for milk was necessitated in cows with clinical metritis. Maximum levels in uterine secretions and predicted concentrations of oxytetracycline in the uterus were lower than MIC values. Systemic administration of long-acting oxytetracycline did not guarantee clinical cure and was not a suitable choice for treatment of clinical metritis associated with *Trueperella pyogenes*. The appropriate approach to antibiotic treatment of uterine infections of cows requires knowledge on penetration of the antibiotics at the site of infection and sensitivity of pathogens.

## 1. Introduction

Among the uterine diseases, clinical metritis is a common complication in dairy farms with financial impact due to increased number of services per conception and decreased milk yield [1]. The costs of treatment and the emergence of resistance to antimicrobial drugs are some serious concerns. Clinical metritis is often associated with mixed infections [2] and isolation of pathogenic bacteria such as *Escherichia coli* and *Trueperella pyogenes* [3]. 

Broad spectrum antibiotics are used for treatment of these mixed infections of the uterus [4]. Several papers attempted to summarize the knowledge about antibiotic use in treatment of endometritis and metritis in cows and to discuss the efficacy of tetracyclines, macrolides, fluoroquinolones and sulfonamide-trimethoprim combinations [5,6,7]. A meta-analysis of the published data reveals that application of ceftiofur decreases the prevalence of metritis although some of research studies reported conflicting results [7,8]. Another problem discussed by Haimerl et al. [7] is related to shortage of data that allow making consistent conclusions on the efficacy of the applied drugs. However, the emergence of resistance to antibiotics restricts the use of cephalosporins and fluoroquinolones in veterinary medicine [9,10]. Therefore, more attention is paid to the prudent use of “old” antibiotics and efforts for establishment of clinical breakpoints have been made [11].

Tetracyclines are widely applied in veterinary practice, including in treatment of uterine infections such as metritis [12]. Published studies reported pharmacokinetics of long-acting oxytetracycline formulations in lactating cows based on plasma and milk concentrations after systemic administration of the antibiotic [13]. Its penetration in plasma and milk after intrauterine administration in cows with metritis was described [14,15]. The disposition in the milk was studied for determination of withdrawal time [16]. Concentrations in the uterus after intramuscular administration of long-acting oxytetracycline formulations in calves at a dose rate of 20 mg kg^−1^ were investigated [17]. Concentrations in the uterine tissues and uterine secretion of oxytetracycline were studied by Masera et al. [18] in healthy cows after intravenous and uterine administration of the antibiotic more than 40 years ago. Uterine tissue inflammation results in increased blood flow to the uterus and in breakdown of epithelial barriers [16]. Therefore, we hypothesize that the disposition of oxytetracycline at the site of infection can be affected by the severity of inflammation. No data about the disposition of tetracycline antibiotics in cows with clinical cases of metritis with simultaneous detection of pathogens causing the infection are available. The contemporary approach to the treatment of bacterial infections requires knowledge about pharmacokinetics and pharmacodynamics of the applied antibiotics. Information about the penetration of the antibacterials at the site of action at effective concentrations is crucial for success of the therapy.

Therefore, the aim of the current investigation was to evaluate the pharmacokinetics of intramuscularly administered oxytetracycline as a long-acting drug dosage form in cows with clinical metritis with special emphasis on its penetration in the uterine secretion. As an integral part of the evaluation of oxytetracycline efficacy, a microbiological assay has been performed for determination of the main bacterial pathogens and their sensitivity to oxytetracycline was studied.

## 2. Results

The animals were diagnosed with clinical metritis, grade 1. The body temperature was within the normal range and only purulent secretion from uterus was observed. The appetite, water consumption and the milk yield were not affected.

### 2.1. Pharmacokinetics of Oxytetracycline

**Figure 1:**
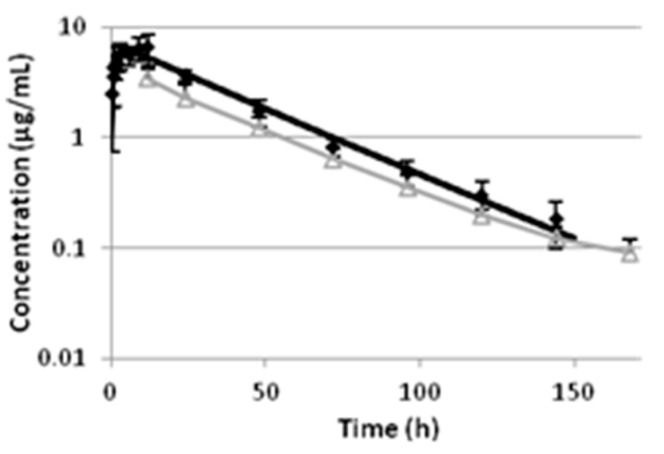
Semi-logarithmic mean ± SD plasma (predicted levels—black line and ♦—observed concentrations) and milk (predicted levels—gray line and Δ—observed concentrations) concentrations of oxytetracycline vs. time curve after a single intramuscular administration in cows (*n* = 6) at a dose rate of 20 mg kg^−1^.

The pharmacokinetic parameters of oxytetracycline following intramuscular administration are presented in Table 1 and on Figure 1. Analysis of the data for plasma by one-compartmental model showed relatively fast absorption with absorption rate constant k_ab_ 0.87 ± 0.51 h^−1^ and absorption half-life t_1/2ab_ of 0.79 ± 0.46 h. The values of the other parameters such as k_el_ (0.03 ± 0.004 h^−1^), T_max_ (4.05 ± 1.80 h), C_max_ (6.21 ± 1.27 µg mL^−1^) and AUC (253.93 ± 34.24 h µg mL^−1^) were very close to those calculated by non-compartmental analysis (Table 1).

The levels of oxytetracycline in the milk were below the limits of quantification (LOQ) at the first two sampling intervals, 0.5 and 0.75 h after the treatment. The first measurable concentration was found 1 h after the treatment. They remained lower than the concentrations in plasma during the entire study (Table 2 and Figure 1). Oxytetracycline levels in milk were lower than the LOQ in three cows 168 h after treatment. The maximum concentrations in milk were significantly lower and were achieved significantly later than the levels in plasma. The elimination half-life for milk and plasma had similar values.

Oxytetracycline was found in the uterine secretion at all sampling intervals (Table 3). High individual variations in the concentrations of the antibiotic were observed between tested cows. The levels of oxytetracycline in the uterine secretion were close to those in plasma 48 h after treatment. The median values of the concentrations in the uterine secretion were twice-lower during the other intervals.

In silico prediction of oxytetracycline levels in the uterine tissue suggested that the antibiotic penetrated in the superficial and deep compartments of the uterus with partition coefficients of P_uterus:pl_ 0.789 and 0.765, respectively.

### 2.2. MIC Concentration of Oxytetracycline against Trueperella pyogenes 

The microbiological assay of uterine secretion revealed presence of *Trueperella pyogenes* (*n* of cows = 4), *Escherichia coli* (*n* = 1) and *Vibrio spp*. (*n* = 1). No pathogenic bacteria were isolated from milk samples. Additionally, the minimum inhibitory concentrations for *Trueperella pyogenes* as a microorganism associated with clinical metritis in cows were determined. Minimum inhibitory concentrations of oxytetracycline against the isolates of *Trueperella pyogenes* were between 16 and 64 μg mL^−1^.

### 2.3. Clinical Outcome after Treatment with Oxytetracycline

Four of the animals were clinically cured. Three cows (No. 1, 2 and 3) were inseminated and pregnancy was diagnosed by means of ultrasound. Less sensitive *Trueperella pyogenes* strains with MIC value of 64 μg mL^−1^ were isolated from cows No. 4 and 5. These animals showed signs of chronic endometritis during the next oestrus and were subjected to treatment. The last animal demonstrated normal clinical oestrus without signs of chronic inflammation of the uterus but it was not inseminated.

## 3. Discussion

The prudent use of antimicrobial drugs for treatment of bacterial infections in farm animals is one of the important limitation steps against selection and spread of resistance. The challenge for practitioners in dairy farms consists of implementing an adequate management program for prevention of diseases and in cases of clinically manifested infections of the genital tract, treating them efficiently to maintain the fertility of the cows. Clinical cases of endometritis and metritis are treated by parenteral administration of antibiotics, most often intramuscularly, or locally by the intrauterine route [7,16]. Oxytetracycline is one of the most often used antibiotics in farm animals, including in cases of infections in the genital tract of dairy cows [16].

Pharmacokinetics of long-acting drug formulations of oxytetracycline in cows has been well described but data after its intramuscular administration in cows with clinical metritis are not published. The long-acting formulation of oxytetracycline, administered at a dose of 20 mg kg^−1^ in the current study, showed similar values of elimination half-life to those of dairy cows treated with other long-acting formulations of the antibiotic at a dose rate of 10–11 mg kg^−1^ [13]. C_max_ values were higher because of the double administered dose and were attained slightly earlier in comparison to the results from the cited study [13]. Half-life of absorption was longer (1.03–1.52 h) when the drug was administered at lower doses [13]. Kumar and Malik [19] found similar pharmacokinetic characteristics in healthy calves as in cows with clinical metritis after single i.m. administration of a long-acting oxytetracycline. The cited authors reported C_max_ of 5.34 ± 0.31 µg mL^−1^ at T_max_ of 8.4 ± 0.4 h, t_1/2el_ of 25.63 ± 1.26 h and AUC of 236.63 ± 0.15 h µg mL^−1^. Altogether these data indicate that the observed pharmacokinetics of long-acting oxytetracycline formulations in cows with clinical metritis is similar to the reported data in healthy dairy cows and calves.

Compared to C_max_ in plasma, oxytetracycline reached significantly earlier twice lower maximum concentrations (*p* < 0.05) in the milk of cows with clinical metritis. Earlier reports show that the observed ratio of free milk to free serum concentration during equilibrium was similar to the calculated ratio in our study [20]. The concentrations in milk between 48 and 144 h after treatment were close to the levels in blood in support of previously reported data [21]. Concentrations in milk in healthy lactating cows after i.m. administration of a long-acting oxytetracycline at a dose of 20 mg kg^−1^ were lower than the values observed by us [22]. The data from our study and previous reports suggest that higher penetration of oxytetracycline in milk can be expected in cows with clinical metritis. Increased blood flow during uterine inflammation can lead to secretion of oxytetracycline into the uterus and to re-absorption of the antibiotic from the uterine secretion into the blood in cows with clinical metritis, especially after administration of long-acting drug dosage forms, and thus to secretion at higher concentrations into the milk [16]. This is a probable reason for high levels of oxytetracycline in the milk, found in the current study if compared to the data in healthy animals. Similar observations, suggesting higher penetration through the blood–milk barrier, were reported for cows with endometritis and cows with metritis after intrauterine administration of oxytetracycline [16]. Nevertheless, the proposed withdrawal time of 7 days by the manufacturer of the used dosage form is long enough and the measured concentrations of oxytetracycline were lower than MRL of 100 µg L^−1^ 168 h after treatment.

Much of the literature is related to investigations in healthy cows and few studies published around 40 years ago present data about the disposition of oxytetracycline after intravenous (i.v.) or i.m. administration in animals with genital tract infection. There is a shortage of evidence for the efficacy of i.m. administration of oxytetracycline for treatment of metritis in cows. The treatment efficacy is highly dependent on the possibility of the antibiotics to reach the site of infection. Penetration of oxytetracycline in healthy cows and in four cows with chronic endometritis after intramuscular administration at a dose of 8 mg/kg as 5% propylene glycol solution has been investigated [18]. In healthy cows, the concentrations in the endometrium were nearly 5 times higher than in plasma 12 h (4.05 ± 1.19 µg g^−1^) and 24 h (2.1 ± 1.3 µg g^−1^) after i.m. treatment. These levels were almost twice-lower in the endometrium of cows with chronic endometritis at the same time intervals: 2.3 ± 1.0 µg g^−1^ and 0.96 ± 0.72 µg g^−1^, respectively. They remained higher in comparison to levels in plasma [18]. The modeling of concentrations in the uterine tissue, performed in our study, predicted comparable penetration in the superficial and deep compartments of uterus. The predicted concentrations in the uterus, based on P_uterus:pl_ coefficient, were between 1.34 µg g^−1^ and 5.0 µg g^−1^ over the first 48 h after treatment. They were close to the measured concentrations reported by Masera et al. [18]. Landoni and Errecalde [17] found 1.1 ± 1.8 to 2.6 ±1 µg g^−1^ oxytetracycline in the uterus of healthy Hereford calves during the first 48 h after treatment with a long-acting formulation at a dose of 20 mg kg^−1^. In another study, similarly to our results, the predicted concentrations in the endometrium and uterine wall after simulation of the penetration of oxytetracycline, administered i.v., twice daily at a dose rate of 11 mg kg^−1^, were lower than the levels in plasma [23]. However, these results require validation with determination of the concentrations after biopsy in cows with clinical metritis. Much higher concentrations were measured in the uterine secretion of individual cows with clinical metritis at different intervals after i.m. treatment with oxytetracycline. In cows No 3 and 5, these levels were between 2 and 9-fold higher than in plasma (at 24 h) which can be related to the severity of tissue inflammation of the uterus. The median values of the uterine secretions/plasma ratio of oxytetracycline, observed in our study, demonstrated twice lower levels in the uterine secretion compared to these in plasma and the range showed that high variations can be expected between individual animals. Masera et al. [18] reported twice-higher mean antibiotic levels in the uterine secretion compared to these in plasma in cows with endometritis. The cited study did not discuss presence of inter-individual variations and absence of the data about the minimum and maximum levels does not allow comparison of the results. Observed concentrations of oxytetracycline in uterine secretion after i.m. administration in cows give evidence for penetration of the antibiotic in the genital tract tissues and secretion through the uterine epithelium which can result in achievement of effective concentrations in the uterus and uterine secretion against some pathogenic bacteria. High inter-individual variation in the uterine secretion levels among the cows in the current study may be attributed not only to the different breeds but also to the different periods for development of postpartum clinical metritis [23]. Altogether these data allow us concluding that oxytetracycline can be secreted through the uterine tissue. 

The cited investigations deal with the data for oxytetracycline penetration in genital tissues but do not provide information about the pathogenic isolates from the same cows causing clinical metritis. Contemporary studies proved that bacteriological tests assist the prudent use of antibiotics, resulting in reduction of the number of cases without clinical cure [24]. Microbiological assays for isolation of a specific pathogen and determination of its MIC values are crucial for selection of the proper antibiotic for therapy of uterine infections [4]. Isolated pathogens from the cows with clinical metritis in the current study are among the commonest bacteria causing uterine infections in cows [25,26]. A special attention was paid to *Trueperella pyogenes*, as a pathogen that often causes uterine infections and mastitis at dairy farms. In a study conducted by Galán-Relaño et al., [3] bimodal MIC distribution was detected for oxytetracycline against *Trueperella pyogenes* and MIC_90_ values of 32 µg mL^−1^ were found out. Another study reported MIC values of oxytetracycline within a wide range between 0.25 to ≥128 µg mL^−1^ [27]. MIC values of 16 and 64 µg mL^−1^ of the isolates from cows with clinical metritis were similar to the reported data. Low sensitivity of *Trueperella pyogenes* was associated with overuse of tetracycline antibiotics in veterinary medicine. Although, according to literature data, *Trueperella pyogenes* was isolated from the milk, the pathogen was not detected in milk samples in our study.

The infections, caused by *Trueperella pyogenes*, are usually treated with ceftiofur or with intrauterinely or intramuscularly administered oxytetracycline [28]. Recent studies show different efficacy of oxytetracycline in treatment of endometritis and metritis in dairy cows. Some clinical investigations found higher first service conception rate in groups treated with oxytetracycline compared to other antibiotics [29]. Other authors reported higher efficacy when intrauterine treatment with oxytetracycline was combined with i.m. administration of ampicillin [12], or when only penicillin was used [2]. Successful treatment of clinical metritis depends on the cellular and humoral local immune response which is a prerequisite for less severe consequences of uterine infections in aged animals than in young cows [25]. Correlation between the prevalence of *E. coli* and *Trueperella pyogenes* in cows with uterine infections and balance of uterine microbiota after treatment can be of significance for cure of the animals with clinical metritis [25].

The results from the current study revealed that oxytetracycline penetrated in the uterine tissue and in the uterine secretions at lower levels than MIC values against *Trueperella pyogenes*. Despite that there are no clinical cut-off value and epidemiological cut-off value for *Trueperella pyogenes,* our data demonstrated that intramuscularly administered oxytetracycline was not an appropriate option for treatment of clinical metritis when this microorganism was isolated as pathogen. Clinical efficacy was not observed in two of the cows and they were treated once intrauterinely with 10% povidone iodine solution. Clinical metritis in other two cows was not associated with isolation of *Trueperella pyogenes* and they were successfully treated with broad spectrum oxytetracycline. Other factors such as immune response, balance of microbiota and higher antibiotic concentrations than MIC values (cow No 3) can contribute to the observed cure of the other two animals from which strains of *Trueperella pyogenes* with MIC of 16 µg mL^−1^ were isolated. A limitation of our study was the number of animals, therefore the conclusion on the efficacy of oxytetracycline in treatment of clinical metritis requires additional clinical trials. Although clinical metritis is associated with isolation of more than one species of microbial pathogens and broad-spectrum antibiotics are expected to be effective, the data from the current study showed that treatment should be based on information about the disposition of the antibiotic at the biophase in the infected animals and the sensitivity of the isolated pathogens.

## 4. Materials and Methods 

### 4.1. Drugs and Reagents

Tetravet LA (Ceva Sante Animale, France) was used for treatment of the animals. The drug was administered at the dose rate, recommended by the producer. Oxytetracycline was applied at a dose of 20 mg kg^−1^ bw.

The used reagents were HPLC grade. Oxytetracycline hydrochloride ≥95% crystalline and doxycycline hyclate with purity ≥98% (Sigma-Aldrich, St. Louis, MO, USA) were used for analytical tests. Acetonitrile CHROMASOLV^®^, HPLC grade, ≥99.9% purity (Sigma-Aldrich,), methanol ≥99.8% purity HiPerSolv CHROMANORM^®^ for HPLC isocratic grade (VWR BDH PROLABO^®^), oxalic acid 98% purity (Sigma Chemical Co., St. Louis, MO, USA), ethylenediaminetetraacetic acid disodium salt dihydrate 99.0–101.0% (Na2H2EDTA × 2H2O, Sigma-Aldrich) and trifluoroacetic acid ReagentPlus^®^, 99% purity (Sigma-Aldrich) were used for preparation of the mobile phase and for extraction of the studied antibiotics from the biological matrices.

### 4.2. Animals

The study was conducted between April 2019 and February 2020 according to the rules of Bulgarian legislation (Ordinance No. 20/1.11.2012 on the minimum requirements for protection and welfare of experimental animals and requirements for use, rearing and/or their delivery, License 151/26.09.2016).

Six lactating cows belonging to Training Experimental Farm of Trakia University, Stara Zagora, Bulgaria from different breeds were included in the study. The animals were housed in the experimental farm. They received feed according to the requirements of the species and water ad libitum. The cows were regularly milked twice daily (7:00 h and 17:00 h). The information about the individual animals is included in Table S1. All of them were diagnosed with clinical metritis after observation on days 5, 10, 15 and 21 after parturition for clinical evidence for metritis. The animals underwent a rectal examination to determine uterine health on days 5, 10, 15 and 21 after parturition. During the examination, the uterus was manipulated transrectally to check the uterine contents and confirm the presence of metritis. Cows with abnormal appearance of the vaginal discharge, reported by the vet in the farm, were subjected to rectal examination to confirm the diagnosis metritis. The animals were diagnosed with clinical metritis grade 1 according to the system of Sheldon et al. [26]. The health of the animals was routinely monitored and they were observed for changes in feed intake, condition and udder filling. They did not show clinical signs for other diseases and the body temperature was within the normal range. The animals were included in the experiment after complete medical check, few days after parturition according to the information in Table S1. The clinical status of the animals, included in the experiment, was checked after the end of the investigation. The animals (*n* = 2) that showed signs of endometritis during the next estrus were subjected to treatment once intrauterinely with 10% povidone iodine solution (Jodouter, Bioveta, Czech Republic).

### 4.3. Experimental Design

The cows diagnosed with metritis were treated once intramuscularly with a long-acting oxytetracycline formulation (Tetravet LA, Ceva Sante Animale, France) at a dose rate of 20 mg kg^−1^ bw according to the manufacturer instructions. Blood samples (5 mL) from the subcutaneous abdominal vein were collected in heparinized tubes (2.5 mL Lithium heparin, FL Medical, Italy) before the treatment. Milk and uterine secretion samples were collected aseptically in sterile tubes for microbiological assessment (10 mL) before drug administration, at the day of the treatment. Uterine secretion samples were obtained after catheterization with a sterile catheter. The milk and uterine secretion samples were immediately transported to the microbiology lab. Plasma, free from antibiotic, was separated from the blood sample after centrifugation at 1500× *g* for 10 min and was frozen at −25 °C until analysis. The animals were treated between 8:00 and 9:00 h in the morning after complete milking. Blood samples were collected via the *vena epigastrica cranialis superficialis* in heparinized tubes (2.5 mL Lithium heparin, FL Medical, Italy). They were withdrawn at 0.5, 0.75, 1, 1.5, 2, 3, 6, 9, 12, 24, 48, 72, 96, 120, 144 and 168 h after treatment to assess plasma oxytetracycline concentration. After collection, blood samples were centrifuged at 1500× g for 10 min, the plasma fraction was transferred in sterile Eppendorf tubes and frozen at −25 °C until HPLC analysis. Milk samples (10 mL) were collected at the same intervals as for blood samples. The cows were completely milked 12, 24, 48, 72, 96, 120, 144 and 168 h after treatment and only milk samples from these intervals were used for further pharmacokinetic analysis. At the other intervals complete milking was not possible and milk samples were used to evaluate the time of appearance of the first measurable concentration in the milk. Samples from uterine secretion were obtained via sterile catheter 6, 24, 48 and 72 h after oxytetracycline administration. All the samples were immediately stored at −25 °C until analysis.

### 4.4. Isolation and Identification of Pathogenic Bacteria

The obtained samples from uterine secretion were seeded on Tryptic soy agar (TSA, HiMedia, India) and MacConkey agar (HiMedia, India) and incubated at aerobic conditions for 24–72 h. TSA was used for isolation of aerobe mesophilic pathogenic bacteria causing metritis in cows and was supplemented with 5% defibrinated sheep blood. Primary identification of the isolates was performed with the following tests: Gram staining, Motility test, Catalase test, Oxidase test and Hugh-Leifson oxidative-fermentative test. Additionally, some specific tests were run, according to the characteristics of the isolates, such as Loffler’s medium with serum (NCIPD, Bulgaria) and CAMP test for *Trueperella pyogenes*, IMViC test for *Enterobacteriaceae spp*., including indole detection, methyl red test, Voges-Proskauer test and citrate utilization. The tests were carried out in accordance with the manufacturer’s instructions and the general rules for aseptic work in the microbiology laboratory [30]. In addition to conventional biochemical tests, a semi-automated system for phenotypic identification with microplates of generation GenIII (BioLog, USA) was used. The plates were incubated under aerobic conditions at 33 °C for 20–24 h.

### 4.5. Minimum Inhibitory Concentration Determination (MIC)

*Trueperella pyogenes* was isolated from most of the investigated cows and its sensitivity to oxytetracycline was tested. MICs of *Trueperella pyogenes* isolates were determined using the micro-dilution method in cation-adjusted Mueller Hinton broth (MHB), according to CLSI Guidelines [31]. Taking into account the growth characteristics of *Trueperella pyogenes*, MHB was supplemented with 2% (vol/vol) lysed horse blood [32]. The plates were incubated for 48 h in a CO_2_-enriched atmosphere. The results were read spectrophotometrically at 620 nm wavelength (Synergy LX Multi-Mode Microplate Reader, BioTek, USA). Each experiment was performed in triplicate with 4 independent replications. 

### 4.6. HPLC Analysis

Oxytetracycline concentrations in plasma, milk and uterine secretion were analyzed by HPLC with PDA detection using a method described by Laczay et al. [33] with minor modifications published by Mileva [34]. An aliquot (150 µL) of plasma samples was placed in 1.5 micro-centrifuge tube spiked with 15 µL internal standard (IS, doxycycline 10 μg/mL) and 19.5 µL trifluoroacetic acid (TFA). Samples were vortexed for 1 min and centrifuged at 10,800 g at 22 °C for 10 min. The supernatant was placed in HPLC vials and 20 µL were injected into the HPLC system. The extraction of oxytetracycline from the uterine secretion was performed with 400 µL of sample spiked with 40 µL IS and 52 µL TFA and the explained steps for plasma were followed. The concentrations in the uterine secretion were determined by using the calibration curve for plasma because it was impossible to obtain enough amount of secretion to prepare a separate curve. Four samples of uterine secretion out of 24 had to be diluted and analyzed again due to very high levels of oxytetracycline.

An aliquot of 500 µL milk was mixed with 50 µL IS and 65 µL TFA by vortexing for 1 min. The samples were centrifuged for 10 min at 10,800× g at 22 °C. The supernatant was transferred to another tube and centrifuged again at 10,800× g at 22 °C for 5 minutes. They were filtered through filter paper (pore size 10–20 μm) after the second centrifugation step. The filtrate from each sample was placed in a HPLC vial and 20 µL were injected into the HPLC system (Thermo Fisher Scientific Inc., USA). A Hypersil Gold column (5 μM, 150 × 4.6 mm) was used at room temperature for separation of tetracycline antibiotics. The analysis was performed with PDA detector (Surveyor, Thermo Fisher Scientific Inc., USA) at wavelength of 345 nm. The HPLC system also included a Surveyor LC Pump Plus and a Surveyor Auto sampler Plus. The mobile phase consisted of acetonitrile, methanol, 0.02 M oxalic acid and 0.02 M Na_2_H_2_EDTA × 2H_2_O (20:15:64:1, *v/v/v/v*). The flow rate was 1.0 mL min^−1^. The retention times were 2.7 min for oxytetracycline and 5.7 min for doxycycline. ChromQuest Chromatography Data System (Thermo Fisher Scientific Inc., USA) was used for peak area integrations.

The developed method was validated for bovine plasma and milk in terms of linearity, intra-day and inter-day precision, recovery, limits of detection (LOD) and quantification (LOQ). The values LOD and LOQ for plasma were 0.05 µg mL^−1^ and 0.15 µg mL^−1^, and for the milk 0.026 µg mL^−1^ and 0.086 µg mL^−1^, respectively. The mean accuracy of the method and mean extraction recovery of oxytetracycline determined in standard solutions in plasma were 95.03% and 97.05%. The same parameters for standard solutions prepared in milk were 95.93% and 91.16%. The mean intra- and inter-day precision (RSD %) values for plasma were 6.38 and 7.55, and for milk: 4.34 and 8.76. The calibration curves for milk and for plasma were built using blank milk and plasma samples, respectively, from untreated cows spiked at 7 different concentrations of oxytetracycline (0.05, 0.2, 0.5, 1, 2.5, 5 and 10 µg mL^−1^). IS was added during preparation of the samples for calibration curves. The method showed good linearity for both matrices: *R^2^* = 0.9987 for plasma and *R^2^* = 0.9996 for milk. The test for lack of fit for plasma (*p* = 0.949) and for milk (*p* = 0.977) confirmed these results. 

### 4.7. Pharmacokinetic Analysis and Prediction of Oxytetracycline Concentrations in the Uterine Tissue

Oxytetracycline plasma concentration vs time curve was described by one-compartmental analysis with absorption (Model 3) and by a non-compartment model using Phoenix 8.1.0.34 software (Certara^®^, Cary, NC, USA). The most suitable model for compartmental analysis was selected according to the lowest value of the Akaike information criterion. One-compartmental analysis has been used to characterize the phase of absorption. Non-compartmental approach based on statistical moment theory was applied for analysis of data for plasma and milk. Individual concentrations of oxytetracycline in the plasma and milk used for pharmacokinetic analysis are presented in Table S2 and Table S3, respectively. Cut-off value for goodness of fit was set at *R^2^* > 0.95. A weighting factor 1/y^2^ was used to improve the fit for data of plasma. The area under the curve (AUC) was calculated by the linear-up log-down rule to the last quantifiable drug concentration-time point (Ct) and infinity. Cut-off values for percent of extrapolation of AUC were settled as <20%. Area under the first moment curve (AUMC_0–∞_) was calculated and mean residence time (MRT_0–∞_) was determined from AUC and AUMC. The elimination rate constants (k_el_) associated with the terminal elimination phase following intramuscular administration was estimated by using linear regression of the terminal phase of the log plasma/milk concentration versus time curve. The mean maximum concentration (C_max_) and time to obtain maximum concentration (T_max_) for plasma, milk and uterine secretion were calculated on the basis of the observed values. 

A model developed by Poulin and Theil [35,36] for non-adipose tissues was used for prediction of oxytetracycline concentrations in the uterine tissue. The following equation describes the relation between drug concentrations in plasma and in tissues:

P_t:p_ = (CF_t_/CF_p_)(fu_p_/fu_t_) (1)

where: fu is the unbound fraction in the plasma (p) or tissue (t), and CF_t_/CF_p_ represents a potential quantitative difference of free concentration between tissues and plasma caused by solubility in lipid and water fractions. Drug specific parameters for oxytetracycline (logPo:w -1.3) [37] and tissue-specific parameters were taken from the literature as described in Haritova and Fink-Gremmels [38]. The values were as follows: fu_p_ 0.7 and fu_t_ 0.82; water content in plasma: 0.91; phospholipid content in plasma: 0.0175 and neutral lipid content in plasma: 0.0017. The values for phospholipid content (0.0008) and neutral lipid content (0.0011) in the uterine tissue [39] and the values for water content (0.845 and 0.82 for the superficial and the deep tissue compartments, respectively) were previously published [40]. 

### 4.8. Statistical Analysis 

The linearity of the calibration curves for milk and plasma was confirmed with test for lack of fit and the curves were linear within the tested range at *p* > 0.05. Pharmacokinetic parameters were presented as arithmetic mean ± SD and as geometric mean ± geometric SD in parenthesis [41]. Normal distribution was assessed by Shapiro–Wilk test. Student’s t-test was used to determine statistically significant differences of pharmacokinetic variables between plasma and milk. Differences were considered significant at *p* < 0.05. The concentrations in the uterine secretion were presented as median and range. The analyses were conducted using Statistica for Windows (STATISTICA for Windows 10.0, StatSoft, Inc., USA).

## 5. Conclusions

Pharmacokinetic study of intramuscularly administered long-acting oxytetracycline (20 mg/kg) in cows with clinical metritis showed that the disposition of the antibiotic at the site of infection does not guarantee achievement of effective concentrations when *Trueperella pyogenes* is isolated from the uterine secretions. The sensitivity of the isolated pathogens should be determined. The choice of the antibiotic and its dosing regimen should be based on analysis of the sensitivity of pathogens and the concentration of the antibiotic in the uterus.

## Figures and Tables

**Table 1:** Pharmacokinetic parameters in cows (*n* = 6) with clinical metritis presented as arithmetic mean ± SD (Geometric mean ± geometric SD) after single intramuscular administration of 20 mg kg^−1^ oxytetracycline hydrochloride as long-acting drug formulation.

Parameters	Units	Mean ± SD (Geometric Mean ± Geometric SD)
Non-compartmental analysis—plasma		
k_el_	h^−1^	0.03 ± 0.004 (0.03 ± 0.004)
t_1/2el_	h	25.79 ± 3.77 (25.57 ± 4.27)
T_max_	h	6.17 ± 3.97 (4.76 ± 1.87)
C_max_	µg mL^−1^	7.31 ± 1.91 (7.10 ± 0.05)
AUC_0-t_	h µg mL^−1^	242.36 ± 31.39 (240.63 ± 33.01)
AUC_0–∞_	h µg mL^−1^	250.43 ± 32.71 (248.62 ± 34.02)
Extrapolation of AUC	%	3.21 ± 0.58 (3.17 ± 0.61)
AUMC_0-t_	h h µg mL^−1^	9236.89 ± 1939.66 (9093.71 ± 1707.74)
MRT	h	36.90 ± 5.49 (36.58 ± 5.88)
Non-compartmental analysis—milk		
k_el_	h^−1^	0.024 ± 0.002 (0.024 ± 0.002)
t_1/2el_	h	29.33 ± 2.97 (29.20 ± 2.92)
T_max_	h	12.00 ± 0.00 (12.00 ± 0.00 *)
C_max_	µg mL^−1^	3.43 ± 0.80 (3.35 ± 0.77 *)
AUC_0-t_	h µg mL^−1^	144.19 ± 32.07 (141.63 ± 28.40)
AUC_0–∞_	Hh µg mL^−1^	147.26 ± 32.11 (144.73 ± 28.50)
Extrapolation of AUC	%	2.14 ± 0.80 (2.03 ± 0.74)
AUMC_0-t_	h h µg mL^−1^	6812.28 ± 1298.36 (6716.54 ± 1219.02)
MRT	h	45.31 ± 3.01 (45.23 ± 2.95)
Non-compartmental analysis—uterine secretion		
T_max_	h	12.00 ± 9.29 (9.52 ± 2.78)
C_max_	µg mL^−1^	12.50 ± 11.39 (8.06 ± 5.08)

^1^ T_max_, time of C_max_; C_max_, maximum plasma or milk levels; k_el_, elimination rate constant; t_1/2el_—elimination half-life; AUC_0–∞_, area under the concentration–time curves from 0 to infinity ∞; AUC_0-t_, area under the concentration–time curves on the basis of measured concentrations during the treatment; AUMC_0-t_, area under the moment curve from the time of dosing to the last measurable concentration; MRT, mean residence time on the basis of the predicted data. * Statistically significant difference between plasma and milk at *p* < 0.05.

**Table 2:** Milk/plasma ratio (Mean ± SD) of oxytetracycline concentrations in cows (*n* = 6) with clinical metritis after single intramuscular administration of 20 mg kg^−1^ oxytetracycline hydrochloride as a long-acting drug formulation.

Time (h)	Milk/Plasma Ratio
12	0.54 ± 0.09
24	0.63 ± 0.20
48	0.74 ± 0.24
72	0.81 ± 0.32
96	0.79 ± 0.37
120	0.70 ± 0.17
144	0.80 ± 0.33
168	0.48 ± 0.43

**Table 3:** Oxytetracycline concentrations in uterine secretion in cows (*n* = 6) with clinical metritis (Median and range in the parenthesis) and uterine secretion/plasma ratio after single intramuscular administration of 20 mg kg^−1^ oxytetracycline hydrochloride as a long-acting drug formulation.

Time (h)	Cow 1	Cow 2	Cow 3	Cow 4	Cow 5	Cow 6	Mean ± SD
Concentration (µgmL^−1^)							
6	4.13	2.33	0.66	3.20	8.43	13.67	3.66 (0.66–13.67)
24	1.79	0.38	29.81	2.26	21.86	1.52	2.03 (0.38–29.81)
48	0.90	0.20	5.03	1.48	20.87	0.73	1.19 (0.20–20.87)
72	0.41	0.15	0.48	3.04	8.90	0.41	0.44 (0.15–8.90)
Uterine secretion/plasma ratio							
6	0.87	0.49	0.12	0.48	1.19	2.88	0.68 (0.12–2.88)
24	0.45	0.10	8.35	0.56	6.17	0.52	0.54 (0.10–8.35)
48	0.64	0.12	2.79	1.03	8.09	0.52	0.84 (0.12–8.09)
72	0.58	0.23	0.57	3.53	8.25	0.53	0.57 (0.23–8.25)

## Supplementary Materials

The following are available online at https://www.mdpi.com/2079-6382/9/7/392/s1, Table S1: Information about cows included in the investigation; Table S2: Measured concentrations of oxytetracycline in plasma of each cow (*n* = 6) after single intramuscular administration of 20 mg kg^−1^ oxytetracycline hydrochloride as a long-acting drug formulation; Table S3: Measured concentrations of oxytetracycline in milk of each cow (*n* = 6) after single intramuscular administration of 20 mg kg^−1^ oxytetracycline hydrochloride as a long-acting drug formulation.
