# Supplementary material for: Oxytetracycline Pharmacokinetics After Intramuscular Administration in Cows with Clinical Metritis Associated with Trueperella Pyogenes Infection
Source: Antibiotics (Basel). 2020 Jul 9;9(7):392. doi: 10.3390/antibiotics9070392 (PMC7400317; doi:10.3390/antibiotics9070392)
Supplement: Supplementary file 1 [file antibiotics-09-00392-s001.pdf]

Table S1. Information about cows, included in the investigation

Cow No	Breed	Body weight (kg)	Age (years)	Days after parturition	Mean daily milkiness (L)
1	Jersey cattle	450	5	18	14
2	Bulgarian black and white cattle	470	9	7	17
3	Bulgarian black and white cattle	500	9	8	18
4	Jersey cattle	310	3	16	15
5	Bulgarian black and white cattle	490	9	18	21
6	Brown cattle	480	11	17	16

Table S2. Measured concentrations of oxytetracycline in plasma of each cow (n=6) after single intramuscular administration of 20 mg.kg-1 oxytetracycline hydrochloride as long acting drug formulation

Time (h)	Cow 1 (μg.mL^{-1})	Cow 2 (μg.mL^{-1})	Cow 3 (μg.mL^{-1})	Cow 4 (μg.mL^{-1})	Cow 5 (μg.mL^{-1})	Cow 6 (μg.mL^{-1})
0.5	1.79	4.13	5.00	0.14	2.17	1.86
0.75	2.40	5.92	5.74	3.18	2.31	2.10
1	2.40	5.07	8.50	4.13	3.25	2.24
1.5	3.63	5.11	4.97	5.02	3.78	2.83
2	4.07	4.80	5.70	5.48	4.74	3.54
3	3.74	5.83	7.02	6.90	6.28	3.60
6	4.77	4.76	5.27	6.62	7.09	4.75
9	6.29	8.83	6.95	5.59	6.51	4.29
12	6.22	10.30	6.60	5.68	5.81	4.44
24	3.97	3.82	3.57	4.08	3.54	2.93
48	1.39	1.73	1.80	1.43	2.58	1.39
72	0.71	0.67	0.83	0.86	1.08	0.77
96	0.48	0.35	0.48	0.44	0.74	0.46
120	0.18	0.27	0.26	0.25	0.46	0.37
144	<LOQ	<LOQ	<LOQ	<LOQ	0.29	0.19
168	<LOQ	<LOQ	<LOQ	<LOQ	<LOQ	<LOQ

Table S3. Measured concentrations oxytetracycline in milk of each cow (n=6) after single intramuscular administration of 20 mg.kg-1 oxytetracycline hydrochloride as long acting drug formulation

Time (h)	Cow 1 (μg.mL^{-1})	Cow 2 (μg.mL^{-1})	Cow 3 (μg.mL^{-1})	Cow 4 (μg.mL^{-1})	Cow 5 (μg.mL^{-1})	Cow 6 (μg.mL^{-1})
0.5	<LOQ	<LOQ	<LOQ	0.02	<LOQ	<LOQ
0.75	0.04	<LOQ	<LOQ	0.04	<LOQ	<LOQ
1	0.05	0.03	0.02	0.27	<LOQ	<LOQ
1.5	0.05	0.04	0.03	0.10	0.12	0.02
2	0.09	0.07	0.05	0.15	0.31	0.05
3	0.39	0.73	0.23	0.29	0.70	0.18
6	1.95	1.90	2.11	2.00	2.29	1.27
9	2.40	3.01	2.48	2.66	3.45	1.85
12	2.81	4.73	3.15	3.80	3.59	2.49
24	2.03	3.46	2.29	1.30	2.67	1.92
48	1.13	1.69	0.85	0.91	1.30	1.44
72	0.56	0.97	0.58	0.56	0.63	0.56
96	0.41	0.52	0.30	0.28	0.34	0.30
120	0.15	0.22	0.22	0.18	0.21	0.20
144	0.08	0.14	0.14	0.12	0.09	0.16
168	0.05	0.10	0.14	0.07	0.08	0.10
